# Magnesium administration provokes motor unit survival, after sciatic nerve injury in neonatal rats

**DOI:** 10.1186/1471-2474-5-33

**Published:** 2004-09-24

**Authors:** N Gougoulias, A Hatzisotiriou, D Kapoukranidou, M Albani

**Affiliations:** 1 Flat 144, Trevose House, Royal Cornwall Hospital, Treslike, Truto- Cornwall, TR1 3LL, United Kingdom; 2 First Neurosurgery Department, AHEPA University Hospital, Thessaloniki, Greece; 3 Dept of Physiology, Medical School, Aristotle University of Thessaloniki, Thessaloniki, Greece

## Abstract

**Background:**

We examined the time course of the functional alterations in two types of muscles following sciatic nerve crush in neonatal rats and the neuroprotective effect of Mg^2+^.

**Methods:**

The nerve crush was performed on the 2^nd ^postnatal day. MgSO_4_*7H_2_O was administered daily for two weeks. Animals were examined for the contractile properties and for the number of motor units of extensor digitorum longus and soleus muscles at three postnatal stages and adulthood. Four experimental groups were included in this study: i) controls, ii) axotomized rats, iii) magnesium treated controls and iv) axotomized and Mg^2+^-treated rats.

**Results:**

Axotomy resulted in 20% MU survival in EDL and 50% in soleus. In contrast, magnesium treatment resulted in a significant motor unit survival (40% survival in EDL and 80% in soleus). The neuroprotective effects of Mg^2+ ^were evident immediately after the Mg^2+^-treatment. Immature EDL and soleus muscles were slow and fatigueable. Soleus gradually became fatigue resistant, whereas, after axotomy, soleus remained fatigueable up to adulthood. EDL gradually became fastcontracting. Tetanic contraction in axotomized EDL was just 3,3% of the control side, compared to 15,2% in Mg^2+^-treated adult rats. The same parameter for axotomized soleus was 12% compared to 97% in Mg^2+^-treated adult rats.

**Conclusions:**

These results demonstrate that motoneuron death occurs mostly within two weeks of axotomy. Magnesium administration rescues motoneurons and increases the number of motor units surviving into adulthood. Fast and slow muscles respond differently to axotomy and to subsequent Mg^2+ ^treatment in vivo.

## Background

In the rat the first 3 weeks of life constitute a critical period of neuromuscular plasticity. Contractile properties of muscles are not inherent, but are determined by the motor nerve, that supplies the muscle. This was shown by experiments of crossinnervation between fast and slow muscles [1].

Axotomy of peripheral nerves in neonatal rats, leads to loss of the bigger immature motoneurons through an excitotoxic process, with bigger neurons firing at a greater frequency. Consequently there is loss of the fast-contracting muscle fibers being innervated by bigger nerve cells [2].

There is much evidence that overactivation of glutamate receptors plays a significant role in this process (glutamate excitotoxicity) [3]. Glutamate is a major neurotransmitter in the CNS. Ionotropic receptors of glutamate (NMDA and AMPA / kainate) have been identified throughout the brain and spinal cord in several species of animals, including humans [4]. Their activation leads to Ca^2+ ^influx into the cell and subsequent activation of a cell death cascade (activation of proteases, lipases and other enzymes leading to cell lysis).

Following neonatal sciatic nerve injury, the surviving motoneurons, take at least 8 days to grow back to the hind limb muscles, whereas most of the motoneurons that die, do so by apoptosis within the first two days [5]. However, it has been shown by previous studies [6,7] that motoneurons are highly vulnerable to excitotoxic cell death, only during the first five days of postnatal life. Sciatic nerve injury by axotomy in neonatal rats has been shown to result in significant reduction in the number of surviving motoneurons in the ventral horn of the lumbar segments. Developing motoneurons become resistant to axon injury when their axon terminals are converted from growing into transmitting structures and form stable connections with the muscle fibers they innervate [8].

Target deprivation from muscle after the 5^th ^postnatal day (P5) does not cause significant motoneuron loss, since damaged axons reinnervate muscle fibers successfully [6,7,9].

In this study we examine the possible neuroprotective effects of systemic administration of magnesium ions, after axotomy in neonatal rats on the 2^nd ^postnatal day. It could be assumed that in our model, magnesium ions penetrate the blood brain barrier, as magnesium has been shown to concentrate in the cerebrospinal fluid after intraperitoneal injection and increase the electrical threshold required to control seizures, in a rat model, by other investigators [10].

We also investigated the time course of motor unit loss and the alterations in the contractile properties of a fast- (EDL) and slow-contracting (soleus) muscle.

## Methods

### Experimental procedures

Wistar Albino rats of both sexes were used in these experiments. All efforts were made in order to minimize the number of animals used and their suffering. The research project complies with the guidelines for animal use, established by the American Physiological Society and was approved by the local ethical committee in accordance with EEC Council Directive 86/609. The day of birth was taken as P0 (zero). At the 2^nd ^postnatal day the sciatic nerve of the left hindlimb was crushed, in order to perform axotomy. Four experimental groups were included in this study: i) control, ii) rats whose sciatic nerve was crushed (axotomy), iii) magnesium treated controls and iv) rats whose sciatic nerve was crushed and were magnesium treated.

The rats were examined for the contractile properties and the motor unit number of two hindlimb muscles (EDL and soleus) at several stages of postnatal development. Tension recordings were held in both hindlimbs (operated and control) in groups of Mg^2+^-treated and non-treated rats at: a) Postnatal day 14 (P14), b) Postnatal day 21 (P21), c) Postnatal day 28 (P28) and d) Adult rats (older than 2 months). In our study six successfully tested Mg^2+^-treated and six non-treated rats of each age-group (48 rats) were included.

### Nerve crush

The animals were anesthetized with ether at the 2^nd ^postnatal day and were operated under sterile conditions. Surgery was performed under an operating stereoscope (10× magnification). The sciatic nerve was identified and crushed at the mid-thigh, just proximal to its division to the tibial and common peroneal nerves. Care was taken not to damage the blood supply to the surrounding tissues. Crush was performed using a fine forceps for 30 seconds. Then the nerve was examined to ensure that the epineural sheath was intact but translucent (axotomy). The wound was closed with fine sutures. All surgical procedures were carried out by the same surgeon. Three hours after wound suturing the neonates were placed back with their mothers.

Specific tests were performed daily in order to confirm the efficacy of axotomy:

a) The plantar- and the dorsi-flexion reflexes were checked: The examiner, placing his index finger on the animals foot, forces it to plantar- and dorsi-flex the ankle. The animal reflects actively, doing the opposite movement, if able to dorsi- and plantar-flex, respectively. No reactive movements are evident after successful denervation.

b) Animals were suspended by their tail and the inability of normal movement of the left hindlimb, indicating successful nerve axotomy, was assessed for the following [11]:

_ Hip and knee in extension

_ Ankle in the operated side in plantar flexion

_ Adduction of the whole limb

_ Weakness of digit extension in operated side

The first behavioral signs of reinnervation after axotomy, should be evident at about 10–12 days after injury, according to other investigators [12].

Only those animals, whose successful axotomy was verified, were included in our study.

### Magnesium administration

The rats of some litters were treated daily with magnesium ions. Mg^2+ ^was injected subcutaneously as a solution of MgSO_4_7H_2_O (0,05 ml of 1 M solution / 10 g body weight). This was the highest tolerable dose. Mortality in higher doses exceeded 80%. The treatment continued until the rats were 14 days old. Control groups were injected daily with the same volume of normal saline (0,05 ml / 10 g body weight). In Mg^2+ ^treated animals weight gain slowed down during the first month of their life. Treated and control adult rats, however, did not show statistically significant differences in their body weight. Axotomized rats, presented with motion deficit on the denervated limb, as described above, for about 2 weeks. After Mg^2+ ^treatment normal kinetic behaviour and reflexes returned on the 7^th ^to 9^th ^postnatal day.

### Tension recording

Animals were anesthetized with Chloral Hydrate (4,5% 1 ml / 100 g of body weight). The EDL and soleus muscles of both the axotomized and the contralateral control leg were prepared. The distal tendons were dissected free and attached to a strain gauge transducer (Dynamometer UFI, Devices) by a short silk suture and the exposed parts of the muscles were kept moist with warm (37°C) Krebs solution (NaCl 118,08 mM, NaHCO_3 _25 mM, glucose 5,55 mM and CaCl_2 _1,89 mM) [13]. The sciatic nerve was exposed. In order to isolate soleus muscle contraction and to avoid summation of concomitant gastrocnemius muscle contraction, after stimulating the sciatic nerve we used to cut the branches innervating the gastrocnemius and plantaris longus muscles. The leg was held rigidly in a position of 90° flexion of the knee and ankle joints, by two pins, inserted in the femoral condyles and the calcaneus, respectively.

The tendons of EDL and soleus muscles were connected to a strain gauge and the tension elicited by sciatic nerve stimulation (Digitimer DS9A stimulator) was displayed on the screen of an oscilloscope (Fluke PM 3380 A). Muscle length was adjusted so as to produce maximal single twitch tension (optimal length), through a micromanipulator allowing motion on the 3 axes (Prior, England). Then stimulus intensity was adjusted in order to elicit maximal tension, using supramaximal (3–9 volts) square pulses of 0,5 msec duration. The signal from the transducer was amplified by a DC transducer amplifier (Neurolog NL 107). Time To Peak (TTP) and Half Relaxation Time (HRT) of the Single Twitch recording were measured. Tetanic contractions were then elicited by stimulating the nerve at 10, 20, 40, 80 and 100 Hz. The duration of stimulus was 250 msec. All devices during the tension-recording procedure were controlled by a pulse programmer (Digitimer D4030).

The number of motor units (MU) was estimated by the incremental method (the number of different single twitch tensions produced by stepwise increments of stimulus intensity). The fatigueability of the muscles was tested by stimulating them at 40 Hz for 250 msec every second. The decrease in tension at 3 minutes of such stimulation was measured and the % Fatigue Index (FI) was calculated:

F.I. = (Initial Tension - Tension at 3 min) / Initial Tension.

After tension recordings were completed, muscles were excised and weighed. The animals were killed by cervical dislocation.

### Statistical analysis

Stiatical analysis of the results was performed using SPSS 10.0 software for Windows. Nonparametric tests (Mann – Whitney for two independent variables and Kruskal – Wallis for more than two independent variables) were used in order to compare data, of different groups. Criterion of statistical significance was set at p < 0.05.

## Results and discussion

### Number of motor units

**Figure 1:**
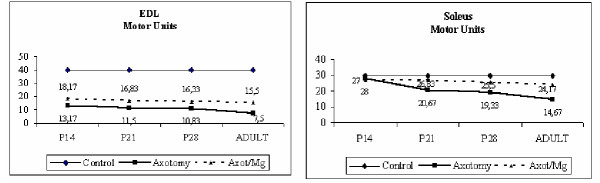
Time course of changes in the number of motor units of EDL and soleus muscles in the left hindlimb. The number of motor units in the right (control) hindlimb is set 40 in the EDL and 30 in the soleus.

**Figure 2:**
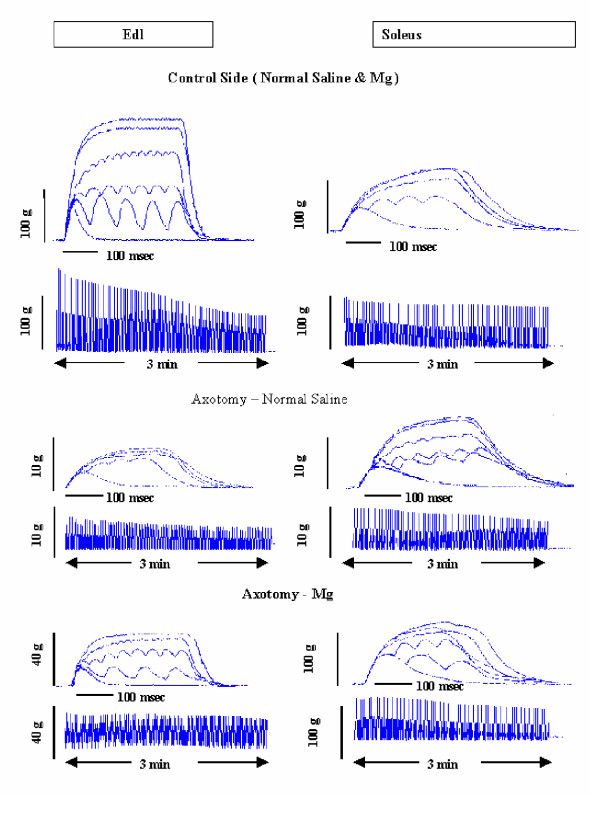
Representative Isometric Tension and Fatigue Recordings of EDL and Soleus in adult rats

The number of motor units was estimated by the stepwise increments of tension, created by stimuli of different intensity. Our findings are in consistency with results of previous work by other authors [14], such as by our previous experience [7]: The control EDL muscle contains approximately 40 motor units, whereas soleus of 30 motor units, independently of animal age (Table 1, Figures 1a and 1b). Treatment with Mg^2+ ^does not affect the number of motor units of control muscles. Axotomy at P2 results in statistically significant (p < 0.05) motor unit loss in both EDL and soleus in all age groups. This means that motoneuron death is already established at P^14^. Treatment with Mg^2+ ^results in a statistically significant difference in the survival of motor units compared to non-treated axotomized rats. It is obvious that the neuroprotective effect of Mg^2+ ^is already established at P14 immediately after the period of treatment (Table 1,2,3 and Figures 1a and 1b). Figure 2, images the different single twitch tension recordings, elicite, in an EDL muscle on the left leg (side of nerve injury), after stimulation of the left sciatic nerve with electrical stimuli of incrementally increasing intensity. In this case, the axotomized EDL muscle consisted of six motor units, that survived axotomy.

### Tension development

Axotomy affects tension development by the muscle (Tables 4 and 5). In adults rats single twitch of EDL is 4.63 ± 0.78% of the control muscle, whereas this of soleus is 16.80 ± 3.03% (Tables 2 and 3). Maximal Tetanic Tension is being developed by stimulation at 100 Hz. Maximal Tetanic Tension in adult rats is only 3.31 ± 0.30% of the control side, whereas after Mg^2+ ^treatment it is 15.16 ± 0.89% respectively. The excessive discrepancy of force outcome ability by both muscles between P28 and adulthood is also noticeable: At P28 respective values are: 18.94 ± 4.16 vs. 40.14 ± 19.34%. At P21 respective values are: 23.35 ± 16.03% vs. 64.22 ± 43.02% (Tables 2 and 3). The absolute values of Tetanic Tension at 100 Hz as listed in Tables 4 and 5, are statistically significant different, between the operated (left) and the control (right) side.

At P14, on the other hand, a tension development deficit does not develop after axotomy.

Maximal Tetanic Tension of soleus is also being reduced after axotomy: 12.44 ± 0.97% of the control side in adult rats. Respective values in the other age groups are: 67.39 ± 39.21% at P28, 79 ± 14.34% at P21 and 81.96 ± 13.56% at P14 (Tables 2 and 3). This marked reduction of tension developing ability of both EDL and soleus, in adult rats, is established after the first month of life. An obvious explanation is the excessive muscle atrophy that occurs gradually as the animal grows up.

Mg^2+ ^treatment, which results in inhibition of muscle atrophy, has been shown to promote motor units survival, and causes the axotomized soleus to remain as strong as the control muscle. Maximal Tetanic Tension of axotomized compared to control side is 97 ± 11.33% in adult rats, 91.45 ± 15.09% at P28, 77.01 ± 25.63% at P21 and 92.12 ± 11.15% at P14 (Tables 2 and 3). Figure 2 shows representative recordings of twitch and titanic tensions, as well as fatigue indexes of EDL and soleus muscles; tables 2, 3, 4 and 5 summarize all the above results.

### Contraction velocity

EDL is normally a fast contracting muscle in adult rats, whereas soleus is a slow one. Immature (P^14^) muscles, however, are not yet differentiated into fast- or slow-contracting. In adult rats TTP and HRT are respectively a) 36 ± 4.4 msec and 28 ± 2.28 msec in the EDL and b) 56.2 ± 3.37 msec and 59.5 ± 3.78 in the soleus (Table 6). At P14 in the EDL, TTP is 56 ± 8.16 msec and HRT is 59 ± 4.34 msec, whereas the respective values for soleus are 74 ± 8.29 msec and 66 ± 8 msec. Axotomy gradually converts EDL into a slow-contracting muscle: TTP is 56 ± 4.97 at P14, 49 ± 10.18 at P21, 43 ± 5.48 at P28 and 77 ± 7.89 in adult rats (Table 6). These values are significantly different to the ones obtained from control muscles. Soleus on the other hand, remains slowcontracting following axotomy, in all age groups. Axotomy does not alter soleus contraction velocity (no statistically significant difference between values).

Mg^2+ ^administration did not affect contraction and relaxation velocity of control muscles (no statistically significant difference). It caused axotomized EDL to become fast-contracting in adult rats (TTP: 38 ± 7.53 and HRT: 43 ± 4.13), as it is predicted for control muscles, whereas soleus' contractility was not affected.

### Fatigueability

Soleus is a fatigue resistant muscle in adult rats, whereas EDL is not. However, both immature EDL and soleus muscles show properties of fatigueable muscles. EDL (control or operated) at P14 is not fatigue resistant, irrespective of Mg^2+ ^administration, with a fatigue index of about 65% (Table 6). During normal development EDL remains fatigable to adulthood, with Mg^2+ ^treatment not affecting this state. Axotomy however, causes EDL to become fatigue resistant in adult rats (F.I = 15.6). Values of operated side compared to those of control muscles, are statistically significantly different even at P21. Mg^2+ ^administration after axotomy does not induce conversion of EDL to a fatigue resistant muscle.

#### Soleus

While soleus is not fatigue resistant at P14 (F.I.= 55.6%), it gradually becomes fatigue resistant during normal development (Table 6). Axotomy on the other hand, results in soleus becoming less fatigue resistant in adult rats (F.I. = 34.7%, statistically significant different than control muscles). However, if axotomy is combined with Mg^2+ ^treatment, the development of soleus into a fatigue resistant muscle is not hindered.

### Muscle weight

Muscle weight on the operated side is statistically significantly reduced compared to the contralateral control side. It is noticeable, however, that there is a marked reduction in muscle weight from P28 to adulthood both in the EDL and the soleus. The reduction in muscle weight of axotomized EDL was already established at P14, whereas in soleus there was a statistically significant difference only after P28 (Tables 4 and 5).

Mg^2+ ^administration provokes muscle weight increase on the operated side. In soleus, values are almost equal to those of control muscles, whereas in EDL the weight gain is less dramatic (Tables 4 and 5).

Our results support findings of previous studies by other workers, concerning the alterations in muscles and motor units after axotomy of peripheral nerves in neonatal rats. In the present study we concentrate on the time course of these alterations. We also focus on the influence of the in vivo administration of magnesium sulphate on motor unit survival and consequently on enhancing force outcome and muscle weight improvement.

Administration of an NMDA or an AMPA receptor antagonist within this critical initial period of development, is thought to reverse the neurotoxic effects of axotomy and results in increased survival of motoneurons. Dizoscilpine malate (MK-801), an NMDA antagonist, has been used in animal models *in vivo *with success, in order to prevent motoneuron death after axotomy [15,16]. However, it was badly tolerated by rats, due to side effects (high mortality).

Magnesium is a non-competitive, voltage dependent, NMDA-receptor antagonist, acting by coupling with the specific Mg^2+ ^site within the pore of the ion channel [17-19]. Its similarity of action compared to MK-801 has been shown in two experimental models of neuropathic pain [20].

Moreover, axotomy in early postnatal period does not only reduce the number of surviving motoneurons and motor units, but also provokes changes in the contractile properties of limb muscles [21]. Immature muscle fibers have not gained yet the characteristics of fast- or slow- contracting type, since not all subtypes of the contractile proteins and enzymes have yet been formed. Extensor Digitorum Longus (EDL), for example, is normally a fast – contracting and fatigable muscle in the adult rat. If axotomy is performed in neonates, EDL becomes slow and fatigue resistant. As other investigators have shown before [22], changes in the contractile properties of immature muscles, during normal development, go on for 30 days after birth, although establishment of mononeuronal innervation of muscle fibres is already fulfilled at P15 and the number of motoneurons innervating a specific muscle is constant after P0.

It has been shown that the number of motor units remains constant after birth [14,23]. This is consistent with our results: The EDL consisted of 40 and the soleus of 30 motor units in all age groups.

It should be mentioned that soleus contractile properties are not significantly altered throughout early postnatal life. Soleus is a slow, non-fatigue resistant muscle at P14 that progressively becomes fatigue resistant. The process of soleus' development into a fatigue resistance muscle is stopped, after sciatic nerve axotomy. Axotomized soleus becomes less fatigue resistant in adult rats, compared to control muscles. However the process of muscle necrosis, as proposed by other authors, could contribute to this result, as well, rather than the loss of motor units alone [25].

Denervated soleus muscle at birth is less fatigue resistant than control muscles in adult rats. This state is already established at P28, with this process progressing further on to adulthood.

As mentioned already, it is noticeable that force outcome even by this 'conservative' muscle is largely affected by axotomy as the animal grows up, after the first month of life. Our data show that even when no marked reduction in the number of motor units occurs, muscle weight does not improve from P28 to adulthood neither does single twitch or maximal tetanic tension. The same phenomenon appears in the EDL as well. The discrepancy seen during the P28 to adulthood interval, both in EDL and soleus, between the reduction in the number of motor units on the one hand, and force outcome (single twitch and tetanic twitch tension) and muscle weight after axotomy on the other, can be explained as a consequence of marked muscle atrophy and necrosis.

As shown by other workers [24], all immature muscle fibers denervated at birth fail to become reinnervate, and the few reinnervated muscle fibers may be overloaded, hypertrophied and eventually necrotized. The final level of tension achieved by the denervated muscle, represents the equilibrium, between decrease in force due to atrophy and necrosis due to regeneration [25]. It has to be considered that nerve injury during early post-natal life, causes permanent changes in the muscles that are not caused by motoneuron death.

Both immature EDL and soleus muscles are slow contracting at P14. During normal development EDL gradually converts into fast contracting, remaining not fatigue resistant throughout life. We found that EDL has already gained characteristics of a fast muscle at P21. Axotomy causing death of the bigger motoneurons, thus destroying the large motor units, converts the muscle into slow-contracting. It was also shown that increase in speed of both EDL and soleus was much reduced after denervation [26].

Our data show that EDL was affected by axotomy much more than soleus. If connection between muscle and nerve is disrupted, as is the case after axotomy, fastcontracting muscles are mostly impaired [12,21,27]. It is known that the overall poorer recovery of immature fast muscles after denervation seems to be due to preferential loss of fibers from fast motor units during reinnervation [28,21,29].

Histological findings [27] and studies on the isometric tension recordings by other workers [21], confirm our findings. However, there is one study [30], suggesting that soleus is the muscle predominately affected after nerve injury.

Previous studies [6] have shown that sciatic nerve crush performed at 5–6 days after birth resulted in a 50% reduction in maximal tetanic tension of the EDL two months after injury, with the muscle becoming more fatigue resistant. Respective values for soleus, however, remained almost equal to those of control muscles. When nerve crush was performed at 3–5 days of life [7], single twitch tension values of the operated side were 36% of those observed for the control side. Respective value for maximal tetanic tension is 50% and for muscle weight is 61%. It is noticeable when comparing those data with our present results, that motoneurons at P2 are much more vulnerable to the excitotoxic effects of axotomy. Axotomy has also been shown to convert EDL into slow-contracting [7,27]. On the other hand repeated injury to the sciatic nerve (at 5 and again at 11 days), has been shown to cause motoneuron death, mostly to the soleus, compared to the tibialis anterior & EDL motoneuron pool, in the spinal cord ventral horn [9].

Magnesium administration by other workers [31] has been shown to cause only a slight improvement in motoneuron survival after nerve crush at birth. However, the same authors found that daily in vivo administration of magnesium sulphate accompanied by NMDA, in axotomized rats at P5, rescues motoneurons destined to die. Our results are strongly suggestive that daily systemic treatment with magnesium sulphate, in order to keep sufficient blood concentrations of magnesium ions, results in increased motor unit survival.

## Conclusions

Our findings strongly support the findings of previous work [31], that has shown magnesium in vivo administration to rescue sciatic motoneurons from cell death, after axotomy.

Local application of magnesium ions to the muscle, by implants affecting achetylcholine release in the neuromuscular junction [32], is suggested to reduce the number of surviving motoneurons by some other mechanism than blocking glutamate receptors. In our study however magnesium ions were not applied directly on the neuromuscular junction. It could be assumed that our results represent the resultant of magnesium ions actions on the nerve and the muscle.

Magnesium administration did not cause any statistically significant influence on the contractile properties of the control muscles in the right unoperated leg, in any age group. Neuroprotection by magnesium reversed the effects of excitotoxicity, predominately in the fast-contracting EDL.

In conclusion, our results show that motoneuron death occurs mostly within two weeks of axotomy, while systemic Mg^2+ ^administration rescues motoneurons and increases the number of motor units surviving into adulthood. Furthermore, fast and slow muscles respond differently to axotomy, as well as, to subsequent in vivo treatment with Mg^2+^.

## Competing interests

The authors declare that they have no competing interests.

## List of abbreviations

AMPA: a-amino-3-hydro-5-methyl-4-isoxazolo-propionic acid

CNS: Central Nervous System

EDL: Extensor Digitorum Longus muscle

FI: Fatigue Index

HRT: Half Relaxation Time

Mg: Magnesium

MU: Motor Units

NMDA: N-methyl-D-aspartate

P: Postnatal

SD: Standard Deviation

TTP: Time to Peak

## Authors' contributions

NG carried out the experiments, participated in the sequence alignment as well as in the design of the study and drafted the manuscript.

AH participated in the experiments and performed the statistical analysis

DK participated in the experiments

MA conceived of the study, and participated in its design and coordination

## Pre-publication history

The pre-publication history for this paper can be accessed here:



## Figures and Tables

**Table 1:** Number of Motor Units

		**Adults**	**P28**	**P21**	**P14**
**EDL**	Controls	**40.00**	**37.83**	**39.50**	**39.33**
		± 1.67	± 1.33	± 1.22	± 1.21
	Axotomy	**7.50**	**10.83**	**11.50**	**13.17**
		± 1.05	± 1.94	± 2.74	± 2.14
	Controls/Magnesium	**39.83**	**41.00**	**38.67**	**37.50**
		± 1.94	± 0.63	± 1.97	± 2.74
	Axotomy/ Magnesium	**15.50**	**16.33**	**16.83**	**18.17**
		± 1.76	± 3.01	± 2.71	± 2.32
**Soleus**	Controls	**29.50**	**30.83**	**29.00**	**31.33**
		± 1.64	± 1.33	± 1.79	± 2.07
	Axotomy	**14.67**	**19.33**	**20.67**	**28.00**
		± 1.75	± 2.66	± 1.63	± 0.89
	Controls/ Magnesium	**30.00**	**29.50**	**28.83**	**29.83**
		± 1.41	± 2.35	± 0.98	± 2.40
	Axotomy/ Magnesium	**24.17**	**25.50**	**26.83**	**27.00**
		± 1.72	± 1.05	± 1.72	± 1.67

Values are expressed as Mean ± SD.

**Table 2:** Muscle Weight, Number of Motor Units, Single Twitch and Maximal Tetanic Tension, expressed as a % ratio, of the operated to the control side, in the EDL, in the four age groups tested. Axotomy has been performed in P2, in all cases

EDL Operated / Control side %				
	
	Single Twitch	Tet-100	Motor Units	Muscle Weight
Adult	4.63	3.31	18.80	10.60
	± 0.78	± 0.30	± 2.93	± 2.62
Adult-Mg	16.59	15.16	38.84	38.88
	± 2.55	± 0.89	± 3.01	± 5.25
P28	28.59	18.94	34.78	45.19
	± 4.58	± 4.16	± 3.03	± 6.29
P28-Mg	38.77	40.14	39.84	64.07
	± 28.78	± 19.34	± 7.30	± 7.22
P21	19.17	23.35	29.22	48.80
	± 10.78	± 16.03	± 7.44	± 6.44
P21-Mg	58.64	64.22	43.73	58.08
	± 36.46	± 43.02	± 8.29	± 29.92
P14	104.99	101.70	33.52	60.91
	± 55.27	± 36.13	± 5.60	± 16.43
P14-Mg	65.94	83.51	48.51	66.11
	± 20.78	± 33.10	18.80	± 13.19

Values are expressed as Mean ± SD

**Table 3:** Muscle Weight, Number of Motor Units, Single Twitch and Maximal Tetanic Tension, expressed as a % ratio, of the operated to the control side, in the soleus, in the four age groups tested. Axotomy has been performed in P2, in all cases.

*Soleus *Operated / Control side %			
	Single Twitch	Tet-100	Motor Units	Muscle Weight

Adult	16.80	12.44	49.87	10.60
	± 3.03	± 0.97	± 6.73	± 2.62
Adult-Mg	87.34	97.00	80.56	38.88
	± 21.06	± 11.33	± 4.34	± 5.25
P28	107.21	67.39	62.77	45.19
	± 53.62	± 39.21	± 8.87	± 6.29
P28-Mg	94.10	91.45	86.67	64.07
	± 25.80	± 15.09	± 5.65	± 7.22
P21	101.74	79.00	71.37	48.80
	± 17.13	± 14.34	± 5.45	± 6.44
P21-Mg	93.49	77.01	93.74	58.08
	± 29.14	± 25.63	± 6.61	± 29.92
P14	90.38	81.96	89.67	60.91
	± 22.35	± 13.56	± 6.41	± 16.43
P14-Mg	89.40	92.12	49.87	66.11
	± 8.04	± 11.15	± 6.73	± 13.19

Values are expressed as Mean ± SD.

**Table 4:** 

**AGE**	**Condition**		**Muscle Weight**	**Single Twitch**	**TET-10**	**TET-20**	**TET-40**	**TET-80**	**TET-100**
**ADULTS**	**CON**	**MEAN**	**0.163**	**67.74**	**92.83**	**117.50**	**172.33**	**212.17**	**259.50**
		*SD*	*0.04*	*11.52*	*1.57*	*18.12*	*1.53*	*17.46*	*22.04*
	**AX**	**MEAN**	**0.017**	**3.08**	**2.87**	**3.16**	**6.39**	**7.67**	**8.54**
		*SD*	*0.003*	*0.28*	*0.53*	*0.49*	*0.72*	*0.69*	*0.60*
	**CON/MG**	**MEAN**	**0.151**	**78.57**	**89.84**	**101.09**	**171.40**	**214.87**	**245.69**
		*SD*	*0.008*	*9.80*	*10.60*	*11.12*	*1.39*	*17.92*	*22.69*
	**AX/MG**	**MEAN**	**0.059**	**12.84**	**13.06**	**15.79**	**19.40**	**27.76**	**37.31**
		*SD*	*0.007*	*0.71*	*0.97*	*1.41*	*1.34*	*2.39*	*4.55*
**P28**	**CON**	**MEAN**	**0.036**	**16.79**	**17.79**	**25.27**	**37.43**	**49.27**	**50.30**
		*SD*	*0.004*	*2.74*	*2.38*	*2.87*	*5.51*	*5.22*	*5.23*
	**AX**	**MEAN**	**0.016**	**3.12**	**3.56**	**5.46**	**8.13**	**9.42**	**9.66**
		*SD*	*0.003*	*1.10*	*1.19*	*2.34*	*2.72*	*2.80*	*2.81*
	**CON/MG**	**MEAN**	**0.016**	**13.39**	**14.08**	**21.90**	**28.24**	**31.29**	**31.61**
		*SD*	*0.001*	*4.94*	*5.32*	*8.73*	*10.16*	*9.49*	*9.20*
	**AX/MG**	**MEAN**	**0.010**	**4.07**	**4.29**	**7.06**	**8.55**	**11.36**	**11.39**
		*SD*	*0.001*	*1.11*	*0.95*	*2.11*	*1.95*	*1.09*	*1.07*
**P21**	**CON**	**MEAN**	**0.021**	**9.09**	**9.64**	**13.98**	**19.00**	**21.75**	**21.65**
		*SD*	*0.006*	*2.01*	*2.11*	*2.85*	*6.64*	*7.87*	*8.57*
	**AX**	**MEAN**	**0.010**	**1.80**	**1.98**	**2.87**	**4.42**	**4.88**	**4.98**
		*SD*	*0.001*	*1.23*	*1.41*	*2.28*	*3.17*	*3.86*	*3.88*
	**CON/MG**	**MEAN**	**0.016**	**6.87**	**8.45**	**13.76**	**17.30**	**19.89**	**20.13**
		*SD*	*0.005*	*2.97*	*4.85*	*7.54*	*9.76*	*12.12*	*13.24*
	**AX/MG**	**MEAN**	**0.008**	**3.39**	**3.72**	**6.47**	**8.42**	**9.42**	**9.47**
		*SD*	*0.002*	*1.24*	*1.34*	*2.47*	*2.43*	*2.85*	*2.90*
**P14**	**CON**	**MEAN**	**0.009**	**4.02**	**5.89**	**7.37**	**9.31**	**10.53**	**10.95**
		*SD*	*0003*	*1.90*	*2.46*	*2.56*	*2.88*	*3.55*	*4.00*
	**AX**	**MEAN**	**0.006**	**3.48**	**4.94**	**7.12**	**8.86**	**10.07**	**10.04**
		*SD*	*0.003*	*0.52*	*1.17*	*1.58*	*1.22*	*1.44*	*1.25*
	**CON/MG**	**MEAN**	**0.007**	**4.41**	**4.67**	**6.54**	**8.79**	**9.63**	**9.94**
		*SD*	*0.002*	*0.83*	*0.87*	*0.73*	*2.10*	*2.59*	*2.59*
	**AX/MG**	**MEAN**	**0.005**	**2.85**	**4.76**	**5.92**	**8.15**	**7.63**	**7.65**
		*SD*	*0.001*	*0.85*	*1.72*	*1.74*	*1.31*	*2.55*	*2.52*

All values are presented in grams. TET-10, -20, -40, -80, -100, means Tetanic Twitch Tension in 10, 20, 40, 80, 100 Hz, respectively.

**Table 5:** 

**AGE**	**Condition**		**Muscle Weight**	**Single Twitch**	**TET-10**	**TET-20**	**TET-40**	**TET-80**	**TET-100**
**ADULTS**	**CON**	**MEAN**	**0.123**	**39.23**	**42.15**	**60.32**	**90,45**	**131.33**	**141.44**
		*SD*	*0.015*	*7.84*	*5.22*	*15.30*	*8,31*	*16.95*	*13.37*
	**AX**	**MEAN**	**0.018**	**6.43**	**7.38**	**8.99**	**15,71**	**17.07**	**17.51**
		*SD*	*0.003*	*0.65*	*0.52*	*0.89*	*1,61*	*1.01*	*0.90*
	**CON/MG**	**MEAN**	**0.106**	**37.05**	**51.00**	**71.75**	**99,32**	**130.48**	**138.02**
		*SD*	*0.012*	*6.67*	*8.28*	*8.00*	*9,95*	*10.00*	*7.83*
	**AX/MG**	**MEAN**	**0.095**	**31.46**	**43.31**	**73.91**	**105,28**	**124.88**	**133.31**
		*SD*	*0.010*	*4.41*	*6.27*	*9.73*	*9,98*	*11.62*	*10.60*
**P28**	**CON**	**MEAN**	**0.029**	**8.45**	**10.37**	**16.45**	**23,42**	**27.34**	**29.07**
		*SD*	*0.005*	*0.86*	*1.90*	*4.12*	*2,91*	*4.20*	*3.54*
	**AX**	**MEAN**	**0.019**	**9.24**	**10.06**	**11.94**	**16,16**	**18.25**	**19.61**
		*SD*	*0.002*	*4.84*	*5.07*	*5.45*	*9,31*	*10.74*	*11.01*
	**CON/MG**	**MEAN**	**0.018**	**6.04**	**9.35**	**13.39**	**18,93**	**20.56**	**21.25**
		*SD*	*0.004*	*1.91*	*2.69*	*2.56*	*1,28*	*3.19*	*3.01*
	**AX/MG**	**MEAN**	**0.014**	**5.37**	**7.09**	**7.81**	**14,11**	**18.73**	**19.35**
		*SD*	*0.002*	*0.77*	*0.95*	*0.51*	*1,97*	*3.86*	*3.57*
**P21**	**CON**	**MEAN**	**0.017**	**10.69**	**11.83**	**16.75**	**27,08**	**31.10**	**30.84**
		*SD*	*0.002*	*1.42*	*1.90*	*2.24*	*3,30*	*4.67*	*4.54*
	**AX**	**MEAN**	**0.016**	**10.73**	**11.23**	**15.22**	**21,99**	**23.85**	**23.88**
		*SD*	*0.001*	*1.22*	*2.49*	*1.90*	*2,91*	*2.60*	*1.75*
	**CON/MG**	**MEAN**	**0.014**	**5.89**	**7.69**	**10.83**	**19,70**	**23.64**	**23.24**
		*SD*	*0.002*	*1.69*	*2.39*	*3.22*	*5,90*	*9.27*	*8.69*
	**AX/MG**	**MEAN**	**0.010**	**5.26**	**6.42**	**12.22**	**14,89**	**16.94**	**17.54**
		*SD*	*0.003*	*1.25*	*2.32*	*3.51*	*4,00*	*4.56*	*4.65*
**P14**	**CON**	**MEAN**	**0.006**	**4.01**	**6.16**	**7.32**	**9,14**	**10.33**	**10.46**
		*SD*	*0.001*	*1.62*	*1.20*	*1.48*	*1,96*	*2.10*	*2.02*
	**AX**	**MEAN**	**0.005**	**3.35**	**5.72**	**6.79**	**7,59**	**8.18**	**8.35**
		*SD*	*0.001*	*0.61*	*0.88*	*0.57*	*0,32*	*0.57*	*0.60*
	**CON/MG**	**MEAN**	**0.006**	**3.61**	**4.16**	**6.37**	**9,16**	**11.15**	**11.35**
		*SD*	*0.002*	*0.86*	*1.16*	*3.10*	*5,05*	*4.85*	*4.62*
	**AX/MG**	**MEAN**	**0.005**	**3.18**	**3.53**	**5.98**	**7,97**	**9.79**	**10.06**
		*SD*	*0.000*	*0.52*	*0.54*	*1.12*	*1,54*	*2.71*	*2.94*

All values are presented in grams. TET-10, -20, -40, -80, -100, means Tetanic Twitch Tension in 10, 20, 40, 80, 100 Hz, respectively.

**Table 6:** Contraction (TTP) – Relaxation (HRT) Time (msec)

	**EDL**							
	**P14**		**P21**		**P28**		**ADULT**	
	TTP	HRT	TTP	HRT	TTP	HRT	TTP	HRT
Control	56 ± 4.97	59 ± 4.34	38 ± 6.45	42 ± 14.61	35 ± 3.93	32 ± 2.34	36 ± 4.40	28 ± 2.28
Axotomy	56 ± 4.97	60 ± 6.25	49 ± 10.18	56 ± 16.26	43 ± 5.48	44 ± 7.92	77 ± 7.89	71 ± 11.50
Control/Mg	54 ± 4.97	56 ± 4.08	40 ± 2.83	34 ± 7.20	44 ± 2.81	38 ± 4.80	40 ± 4.90	34 ± 3.20
Axotomy/Mg	56 ± 4.49	62 ± 4.80	49 ± 3.72	46 ± 10.61	43 ± 3.72	41 ± 7.34	38 ± 7.53	43 ± 4.13
	**SOLEUS**							
	**P14**		**P21**		**P28**		**ADULT**	
	TTP	HRT	TTP	HRT	TTP	HRT	TTP	HRT

Control	74 ± 8.29	66 ± 8.00	58 ± 5.22	64 ± 5.85	48 ± 3.25	58 ± 3.70	56 ± 3.37	60 ± 3.78
Axotomy	78 ± 6.81	75 ± 5.75	60 ± 9.77	63 ± 7.39	51 ± 7.45	58 ± 10.90	58 ± 5.99	61 ± 7.23
Control/Mg	75 ± 5.90	76 ± 6.62	51 ± 14.72	53 ± 27.62	49 ± 13.20	61 ± 13.41	56 ± 5.47	60 ± 7.55
Axotomy/Mg	73 ± 6.54	72 ± 6.12	54 ± 2.53	47 ± 4.34	59 ± 5.22	53 ± 8.14	61 ± 2.00	60 ± 3.77

Values are expressed as Mean ± SD.

**Table 7:** Fatigue Index

		Fatigue Index %			
		AGE			
Muscle	Condition	P14	P21	P28	ADULT
EDL	Control	65.4	61.4	75.1	55.0
	Axotomy	57.2	44.7	41.6	15.6
	Con/Mg	72.4	72.4	63.1	56.4
	Axot/Mg	65.2	40.6	34.2	9.9
Soleus	Control	55.6	42.0	35.8	17.8
	Axotomy	68.3	42.7	44.8	34.7
	Con/Mg	67.1	34.5	30.6	19.1
	Axot/Mg	64.9	54.0	58.9	19.8

Values are expressed as Mean ± SD.
